# Phytotoxic and genotoxic effect of Aluminum to date palm (*Phoenix dactylifera* L.) *in vitro* cultures

**DOI:** 10.1186/s43141-019-0007-2

**Published:** 2019-10-21

**Authors:** Khairullah M. Awad, Ansam M. Salih, Yahya Khalaf, Aqeel A. Suhim, Mohammed Hamza Abass

**Affiliations:** 1 Date Palm Research Centre, University of Basra, Basra, Iraq; 2 Plant Protection Department, College of Agriculture, University of Basra, Basra, 61001 Iraq

**Keywords:** Aluminum, biochemical analysis, date palm, pollution, protein patterns

## Abstract

**Background:**

Al is a common metallic element found in earth's crust and is a toxic pollutant present at high concentrations in acidic soil, thus affecting plant growth. Despite being well studied as a toxic element, the effects of Al on date palm have not been investigated. This study aimed to assess the toxic effects of different Al concentrations on the development and growth of date palm callus and evaluate the biochemical and molecular response of date palm cells under Al stress.

**Results:**

Our study revealed the phytotoxicity of Al concentrations (50, 100, 150 and 200 mg.l^-1^) on date palm callus. The fresh and dry weight and the number of produced embryos were significantly decreased in response to Al concentration. At 150 mg.l^-1^, the embryo number decreased to 1.66 compared with the 19.33 in the control treatment. At high Al concentration (200 mg.l^-1^), the callus failed to produce any embryo. Biochemical analysis revealed that Al exposure had negative effect on callus. Total soluble carbohydrates, total soluble protein and free amino acids were decreased in plants receiving 200 mg.l^-1^ Al treatment compared with those in the untreated ones. A similar decline was observed in total soluble protein and free amino acid in response to Al treatment. Significant accumulations of malondialdehyde, H_2_O_2_ and peroxidase activity accompanied the increase in Al concentration in cultured tissues, revealing the generation of toxic reactive oxygen species in affected cultures. The genotoxic effect of Al at high concentrations (150 and 200 mg.l^-1^) was revealed by protein patterns.

**Conclusion:**

Our findings revealed for the first time the phytotoxicity of Al to date palm callus. At 200 mg.l^-1^, Al prevented the embryo production of date palm callus. At 50, 100, 150 and 200 mg.l^-1^, Al negatively affected the biochemical characteristics of date palm callus. At 150 and 200 mg.l^-1^, Al induced changes in protein expression. These data showed that the tissue culture technique can be used as a valuable approach in heavy metal toxicity studies.

## Background

Soil acidification is a result of industrial and agricultural activities that lead to the accumulation of toxic ions, including Al, Zn, Cu, Pb and Cd [7]. Al is the third most abundant element in the earth’s crust but is not considered as an essential nutrient; however, an increased plant growth is observed in soils with low Al concentrations [37]. In soil pH of 5.5 or lower, Al is a toxic factor that limits crop growth and productivity [23, 27]. Al toxicity has several consequences of, including root growth inhibition, oxidative stress as a result of reactive oxygen species (ROS) generation, alteration of cell wall and plasma membrane characteristics, nutrient unbalances, cytoplasmic Ca^2 +^ efflux and induction of callose (1,3- β -D-glucan) formation [26, 37, 38, 41]. The use of *in vitro* tissue culture technique is suitable to study the physiological effects of Al and allows the application of cells with uniform growth and the investigation of physiological and biochemical Al toxicity at the cellular level [20, 39, 45]. The negative effects of Al toxicity in cultured cells for some plant species, such as tomato [28], tobacco [50], wheat [11], *Citrus* species [46] and *Lobelia chinensis* [18], have been investigated. Al toxicity inhibits cell division and elongation, reduces cell growth and decreases callus fresh and dry mass. Al also has deleterious effects on the genomic stability of cells cultured *in vitro* [37]; hence, DNA is the first target of Al toxicity in plant systems because of its presence in the cytoplasm and nucleus of coffee cell protoplast. Furthermore, DNA degradation and cell growth inhibition have been observed, [15] indicating that Al at high concentrations negatively affects double helix rigidity and thus reduces DNA replication.

Al induces protein expression alteration and changes protein profile by inducing the up- and down-regulation of proteins with different expression patterns as revealed by the appearance and disappearance of protein bands in SDS PAGE [12, 44, 46]. Date palm *Phoenix dactylifera* L. belongs to the Arecaceae family and is cultivated mainly for their nutritive fruits [3]. This tree is propagated through seeds, offshoots and tissue culture technology [52]. With tissue culture approach, date palm can propagate by two main methods, namely, somatic embryogenesis and auxiliary bud formation [1, 5]. *In vitro* date palm tissue culture at callus induction stage is used to induce and increase total steroids by adding some heavy metals (Cd and Al) to basal nutrient medium, [53] thereby substantially increasing the total steroid content of date palm callus compared with that of the control treatment.

Al toxicity on date palms have been not been investigated either on whole plants or at the cellular level. Thus, this study was designed to assess the effects of different Al concentrations on the development and growth of date palm callus and evaluate the biochemical and molecular responses of date palm cells under Al stress.

## Methods

### Prepare culture medium

Culture medium was prepared with concentration 4.33 gm. L^-1^ from MS salts [31] provided from Zist Arman Sabz company, supplemented with additions presented in Table 1, then the medium pH was adjusted to 5.8 with NaOH (1N) and autoclaved, after that Aluminum (Al) was at different concentrations, the experimental treatments as follow:

1. Al_0_ as control treatment
2. Al_50_ which Al added at 50 mg.l^-1^
3. Al_100_ which Al added at 100 mg.l^-1^ .
4. Al_150_ which Al added at 150 mg.l^-1^ .
5. Al_200_ which Al added at 200 mg.l^-1^ .

**Table 1:** Additions supplemented to culture medium of date palm callus

Addition	Concentration g.l^-1^
Sucrose	30
Na_2_H_2_PO_4_	170
Myo-inositol	125
Glutamine	200
Thiamine	5
Nicotinic acid	1
Pyridoxine-HCl	1
2,4-D	50
2iP	3
Agar	7
Activated charcoal	1.5

Al was supplemented as AlCl_3_ with micro filters to avoid microbial pollution. The culture medium was poured in test tubes and closed tightly with cotton and then wrapped with aluminum foil.

### Plant materials

Embryogenesis callus of date palm derived from date palm shoot tips of Hillawi cultivar, provided from tissue culture laboratory in Date Palm Research Centre- Basrah University, cultured into medium already prepared with 50 mg as initial weight, callus inoculated into medium in test tubes and incubated with conditions, 25±2° C in a dark culture room for 12 weeks, callus subjected to re-culture every four weeks**.**

After incubation period, Al toxicity was evaluated by following characteristics:

### Fresh and dry weight and embryo numbers of date palm callus

Fresh and dry weight was measured after incubation period; also counted the number of embryos was generated on date palm callus.

### Total Soluble carbohydrates

Date palm callus content of total soluble carbohydrates was estimated depended on Anthrone (97%, Sigma Aldrich, USA) reaction according to [47], the absorbance was measured at 620 nm and glucose was used to prepared standard curve.

### Total soluble protein

[8] Protocol was followed to measure the total soluble protein; Albumin was used as standard curve and absorbance measured at 595 nm.

### Free amino acids

Procedure of [25] was followed to estimate the free amino acids and optical density was measured at 570 nm.

### Malondialdehyde (MDA)

MDA was quantified as a marker of membrane lipid peroxidation, MDA was extracted 5 % (w/v) with trichlotoacetic acid (TCA) (99%, Himedia, India), the absorbance at 532 and 600 nm was used, a calculation of MDA content was done depending on extinction coefficient of 155 [17].

### Hydrogen Peroxide (H_2_O_2_)

H_2_O_2_ content was measured calorimetrically at 390 nm according to [40], H_2_O_2_ (38%, Evonik, Germany) was used to create a standard curve.

### Peroxidase enzyme activity

Procedure of [22] was used to estimate peroxidase activity (U/min/g) depending on the variation of absorption at 470 nm as a result of tetraguaiacol production.

### SDS PAGE electrophoresis

Isolate protein were applied to sodium dodecyl sulphate polyacrylamide gel electrophoresis (SDS PAGE), under non-denaturing procedure as described in [24]. The procedure of [29] to stain and destain the gel with commassie brilliant blue was followed. Electrophoretic stacking was performed using 4% polyacrylamide gels and 10% for electrophoretic separation at 4 ° C for 7 h. Promega (10-225 KDa) was used as protein molecular weight marker, fragments photographed under UV light, the detection of fragments molecular weights was performed using the PhotoCapt MW software 10.0 (Vilber Loumart).

The binary matrix was created according to the fragments present (1) or absent (0); and equation of [32] was followed to measure the genetic similarity index (GSI) as:

$$ GSI=\frac{2A}{B+C} $$

where (A) number of similar fragments in both treatments, (B) and (C) total number of bands in the first and second treatments.

$$ Genetic\ distance=1- GSI $$

Where (GSI) genetic similarity index as explained in the equation above.

The similarity index was used to produce the dendrogram using the unweighted pair group mean average (UPGMA) method [42].

### Statistical analysis

The complete randomized design was used. The obtained data was analyzed with one way analysis of variance (ANOVA), the mean treatments were compared with Least Significant Difference (LSD) test at the probability level of 0.01, statistical analysis was done by using the SPSS-22 statistical software (SPSS In., Chicago, IL., USA) version 22.

## Results

### The effect of Al treatments on fresh, dry weight and embryo numbers of date palm callus

**Fig. 1:**
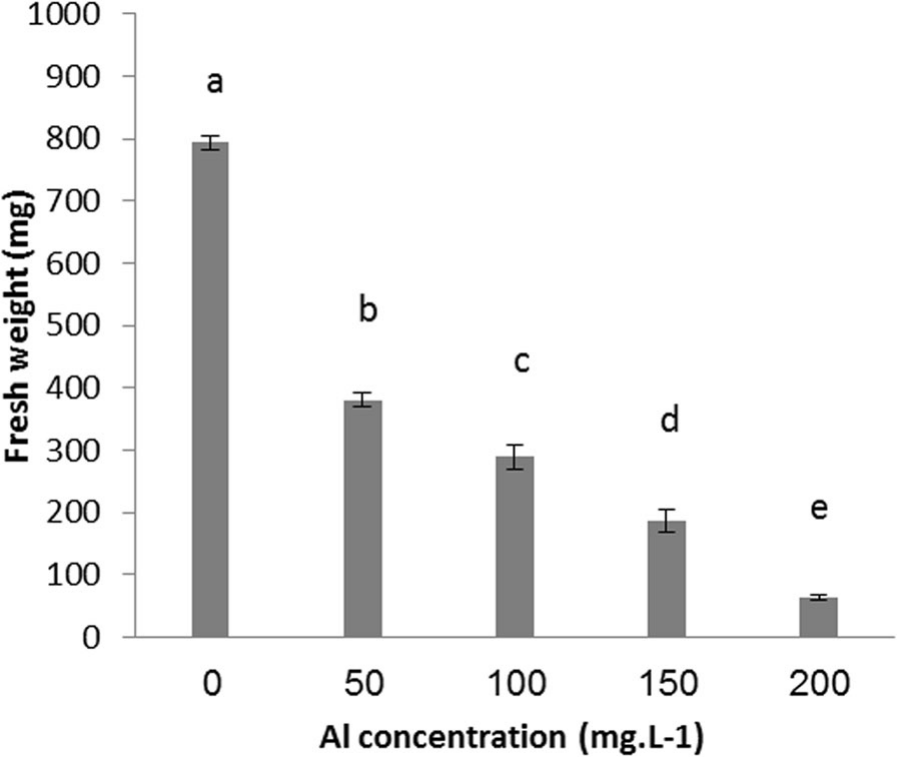
Al toxicity on fresh weight of date palm callus

**Fig. 2:**
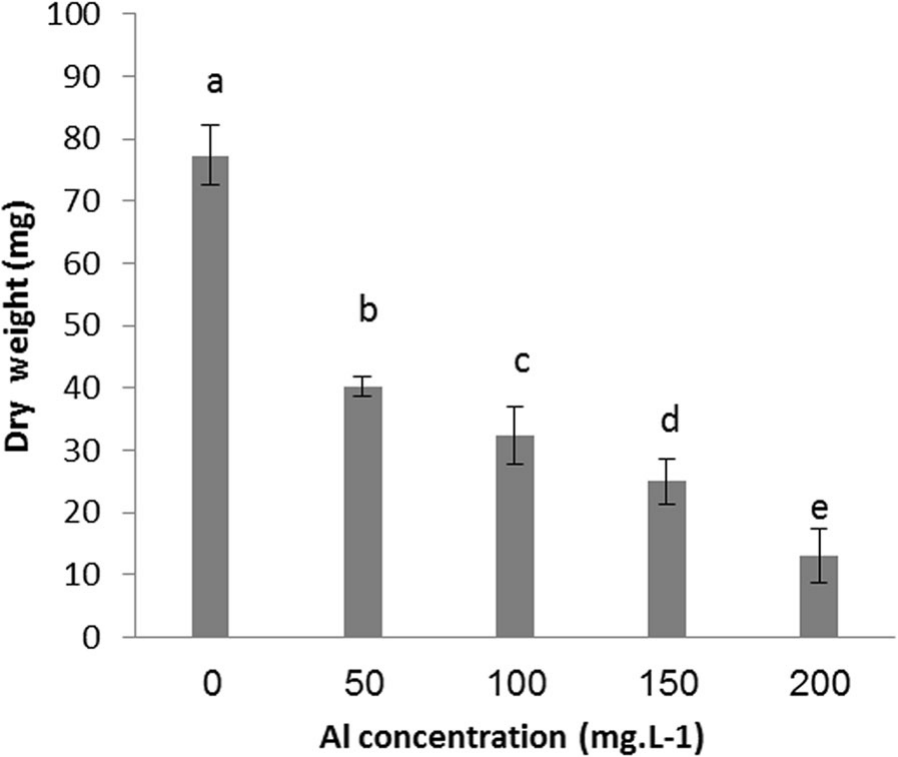
Al toxicity on dry weight of date palm callus

**Fig. 3:**
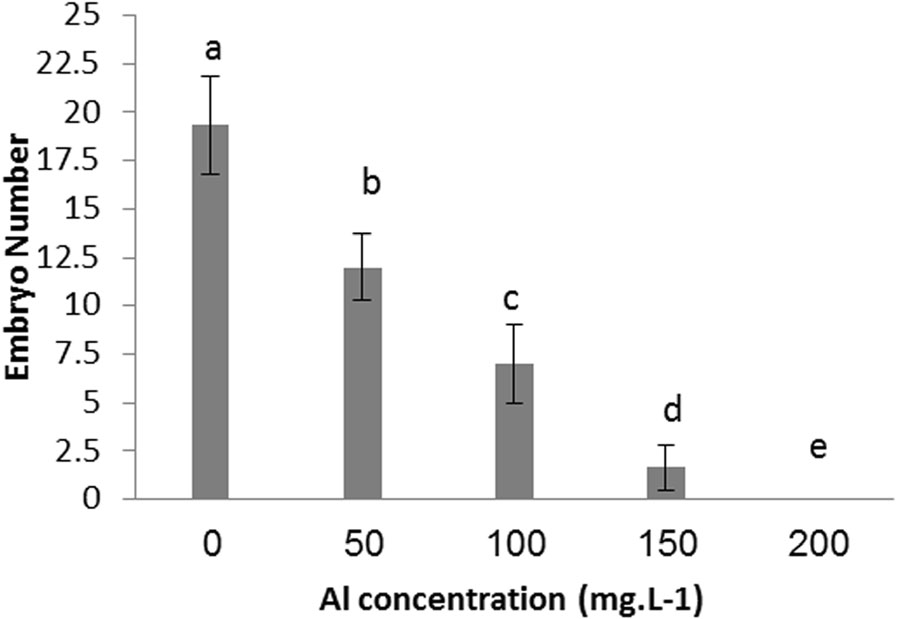
Al toxicity on embryo number of date palm callus

Results presented in Figs. 1 and 2 revealed the toxic effect of Al at a range of concentrations on both fresh and dry weight of date palm callus, Fig. 1 showed that the fresh weight of date palm callus was reached 794.33 mg in control treatment after 12 weeks, while the fresh weight was decreased significantly to 380.33, 288.66, 180.66 and 63.00 mg when callus propagated in medium contain Al at 50,100,150 and 200 mg.l^-1^ respectively. Dry weight of callus was affected when exposed to Al at all investigated concentrations; the highest value of dry weight was recorded in control treatment which was 77.33 mg, while the lowest was recorded in callus exposed to Al at 200 mg.l^-1^ which was 13.00 mg. Results in Fig. 3 showed the dry weight was 40.33, 32.33 and 25.00 mg in Al at 50,100 and 150 mg.l^-1^ treatments, respectively.

Regarding the embryos number, the results illustrated in Fig. 3 showed, the callus treated with Al at 200 mg.l^-1^ did not produced any embryo after 12 weeks, while the embryos numbers were 12, 7 and 1.66 when callus of date palm cultured on a medium supplemented with Al at 50, 100 and 150 mg.l^-1^ respectively, it is noted from results the control treatment produced 19.33 embryos.

### Biochemical responses of date palm callus to Al treatments

The biochemical characteristics of date palm callus under Al treatments were evaluated after 12 weeks, exposure to Al resulted in a significant reduction in carbohydrates, proteins and free amino acids content, in contrast a significant increase in MDA, H2O2 and peroxidase enzyme activity. The results in Table 2 showed a significant effect of Al treatments on total soluble carbohydrates content in date palm callus in comparison to unexposed callus; the highest level of carbohydrates was recorded in control treatment which was 5.87 mg.g^-1^ while Al treatment at 200 mg.g^-1^ produced the lowest carbohydrate level 0.54 mg.g^-1^. Similar reduction was observed with protein content, Al at 200 mg.l^-1^ declined total soluble protein content from 3.39 mg.g^-1^ in control treatment to 0.27 mg.g^-1^ , while the protein content were 1.82, 1.23 and 0.78 mg.g^-1^ when callus exposed to Al at 50, 100 and 150 mg.l^-1^ respectively. Free amino acids content decreased significantly with Al concentration increase and it was evident in callus exposed to Al at 200 mg.l^-1^, the free amino acids content in control treatment was 1.66 mg.g^-1^ reduced to 0.16 mg.g^-1^ in Al at 200 mg.l^-1^ treatment.

**Table 2:** Carbohydrates, protein, free amino acids, malondialdehyde, H2O2 contents and peroxidase activity of date palm callus in response to Al stress

Al concentration (mg.l^-1^)	Total soluble carbohydrates (mg.g^-1^)	Total soluble protein (mg.g^-1^)	Free amino acids (mg.g^-1^)	MDA (nmole.g^-1^)	H_2_O_2_ (μM.g^-1^)	Peroxidase (unit.g^-1^.min.^-1^)
0	5.87± 0.36^*^	3.39 ± 0.08	1.66 ± 0.07	0.46 ± 0.05	0.10 ±0.01	8.80 ± 0.06
50	3.85 ± 1.92	1.82 ± 0.04	0.80 ± 0.05	0.56 ± 0.04	0.12 ± 0.01	9.65 ± 0.12
100	1.97 ± 0.08	1.23 ± 0.05	0.60 ± 0.03	0.60 ± 0.02	0.25 ± 0.04	10.81 ± 0.14
150	1.25 ± 0.06	0.78 ± 0.07	0.41 ± 0.05	0.71 ± 0.04	0.38 ± 0.02	11.89 ± 0.04
200	0.54 ± 0.06	0.27 ± 0.07	0.16 ± 0.02	0.79 ± 0.05	0.52 ± 0.03	12.66 ± 0.21
LSD	1.60	0.11	0.10	0.07	0.30	0.24

^*^Each value represent mean of triplicate ± SD

Opposite trend of results was seen with Malondialdehyde (MDA), the obtained results revealed MDA content was increased in treated callus with Al, thus, was evident by the increase of MDA content from 0.46 nmole.g^-1^ in control to 0.56, 0.60, 0.71 and 0.79 nmole.g^-1^ for Al treatments at 50, 100, 150 and 200 mg.l^-1^ respectively. Results showed that, Al treatments (50, 100 and 150 mg.l^-1^) had no effect on H_2_O_2_ production in treated calli in contrast with untreated callus (0.10 μM.g^-1^); while Al at 200 mg.l^-1^ led to a significant increase in H_2_O_2_ production which reached to 0.52 μM.g^-1^. Peroxidase was noted to be increased significantly as a response to Al treatments, peroxidase activity was 8.8 unit.g^-1^.min.^-1^ in untreated calli, and the activity reached maximum value (12.66 unit.g^-1^.min.^-1^) in Al at concentration of 200 mg.l^-1^. Interestingly, all Al treatments (50, 100 and 150 mg.l^-1^) led to a significant activity of peroxidase compared with control one.

### Protein analysis

**Fig. 4:**
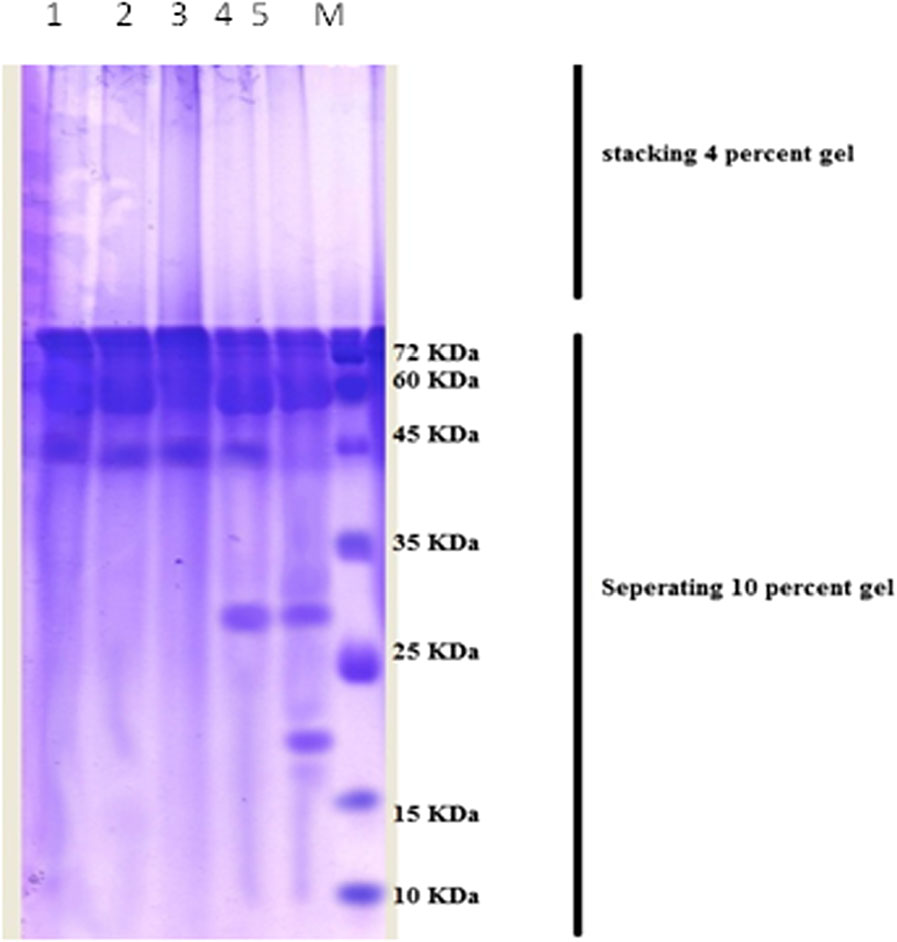
SDS-PAGE profile of date palm callus treated with at different concentrations. M: Protein molecular marker; 1: control treatment; 2: Al at 50 mg.l^-1^ ; 3: Al at 100 mg.l^-1^; 4: Al at 150 mg.l^-1^; 5: Al at 200 mg.l^-1^

SDS-PAGE analysis of protein pattern from date palm callus treated with different concentrations of Al compared to control treatment (Fig. 4, Table 3) revealed that, no difference of protein pattern for Al treatments at 50, 100 mg.l^-1^ compared to control treatment, which produced three fragments at sizes of 77, 54 and 42 KDa as molecular weight. A small difference in Al treatment at 150 mg.l^-1^ was observed, in comparison with control treatment, newly appeared polypeptide with the size of 29 KDa was seen. The major difference distinguished with increase Al to 200 mg.l^-1^, two new expressed polypeptides with the sizes of 29 and 29 KD were observed a, interestingly, fragment with the size 42 KDa was disappeared compared to control treatment.

**Table 3:** Protein profile (presence and absence) in callus of date palm treated with Al (mg.l^-1^) obtained by SDS-PAGE technique

Lane number of band	Molecular weight of band (KDa)	0	50	100	150	200
1	77	1	1	1	1	1
2	54	1	1	1	1	1
3	42	1	1	1	1	0
4	29	0	0	0	1	1
5	19	0	0	0	0	1
Total number of bands		3	3	3	4	4

The results of Table 4 represent the genetic similarities index (GSI) values according to presence and absence of fragments, the results showed the highest GSI value was observed in control treatment and Al treatments at 50 and 100 mg.l^-1^ (100%), while it was (75%) between control and Al at 150 mg.l^-1^, the lowest GSI value was reported in control treatment and Al treatment at 200 mg.l^-1^ (50%).

**Table 4:** Similarity indices according to Nei and Li's coefficients of treated date palm callus with several concentrations of Al obtained by SDS-PAGE electrophoresis

Al Concentration mg.l^-1^	0	50	100	150	200
0	1	1	1	0.75	0.5
50	1	1	1	0.75	0.5
100	1	1	1	0.75	0.5
150	0.75	0.75	0.75	1	0.5
200	0.50	0.50	0.50	0.75	1

**Fig. 5:**
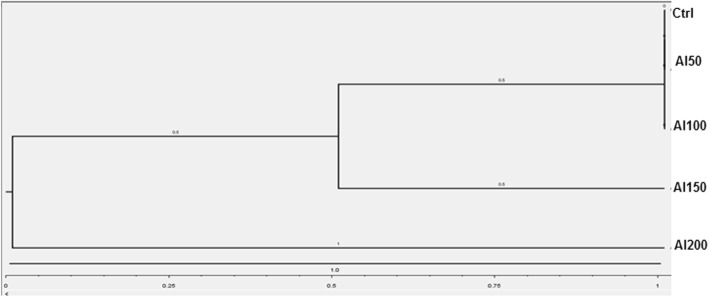
Dendrograms generated by UPGMA cluster method based on Protein SDS-PAGE electrophoresis Ctrl: Control, Al50: Al at 50 mg/L^-1^, Aa100: Al at mg/L^-1^, Al150: Al at 150 mg/L^-1^, Al200: Al at 200 mg/L^-1\^

The dendrogram was created according to genetic distance index (Fig. 5) of protein profile, the cluster grouping showed that all treatments were separated into three clusters, the first included Al treatments at 50, 100 mg.l^-1^ and control treatment, while the second included only Al treatment at 150 mg.l^-1^, while Al at 200 mg.l^-1^ was separated in the third cluster.

## Discussion

Results showed that Al at all examined concentrations significantly affected date palm callus growth. Our findings were based on fresh and dry weight. Al supplemented to medium at 50, 100, 150 and 200 mg.l^-1^ concentrations reduced the fresh weight of callus by approximately 52.11%, 63.65%, 77.25% and 92.06%, respectively, and that of dry weight by approximately 47.84%, 58.19%, 67.67% and 83.18 %, respectively, compared with that of the control treatment. The results in Fig. 3 show that the number of embryos produced on the date palm callus significantly decreased with increasing Al concentration. Furthermore, Al at 200 mg.l^-1^ prevented date palm callus to produce any embryo.

The phytotoxicity of Al at the examined concentrations could be attributed to the inhibition of cell elongation ad division at high concentration (200 mg.l^-1^) but only cell elongation at low concentration [27]. Low Al concentrations induce endogenous nitric acid production and may inhibit cell elongation [16]. In addition, the presence of Al ion in medium limits the transport of many nutrients and blocks their contribution in the metabolic system [33]. High Al concentrations decrease the uptake of nutrients, such as Mg, Ca, P, K, Zn and Fe [13]. The inhibition effect of Al on elongation and cell death might involve two phases; the early phase is distinguished by low sugar uptake and inhibition elongation, and the later phase is involved in ROS generation, eventually leading to cell death [4]. ROS production, respiration inhibition and ATP depletion are important events of Al toxicity in plant cells [49].

Our results in Table 2 indicate a significant decrease in the content of total soluble carbohydrates, total soluble protein and free amino acids of date palm callus, and this trend is inversely proportional to the increase in Al concentration. ATP depletion reduced the energy supply needed for protein synthesis, which can explain the reduction in protein level and free amino acids. Al reduces the total soluble proteins in sorghum plants [10]. In this study, the date palm callus showed significantly increased MDA and H_2_O_2_ level and increased peroxidase activity. Lipid peroxidation is of the first symptoms of Al toxicity in plant cells [34, 49, 51]. The MDA content in date palm callus treated with Al at 200 mg.l^-1^ was increased by up to 1.71-fold compared with that in the control callus. Lipid peroxidation was also induced under Al stress [14, 19, 27, 35]. The increase in lipid peroxidation may be attributed to the binding of Al to biomembrane and leads to rigidity, which in turns causes the generation of radical chain reactions by Fe ions [21, 48]. H_2_O_2_ accumulation in date palm callus was increased up to 5.2-fold when exposed to Al compared with that in the control treatment. Al triggers the production of ROS, including O_2_^-^ [9]. The enzyme super oxide dismutase (SOD) catalyses the dismutation of O_2_^-^ into H_2_O_2_ as a fairly stable form of ROS and O_2_ [6]. The increase in H_2_O_2_ level in date palm callus under Al treatments may be correlated with the increased SOD activity.

The results showed substantially increased peroxidase (POD) enzyme activity for the plants exposed to all experimented Al concentrations. The highest level was observed in date palm callus grown at medium containing 200 mg.l^-1^ Al, showing 1.43-fold more enzyme activity compared with the control treatment. Different genes encoding peroxidase enzymes were expressed under Al exposure in *Arabidopsis thaliana* after 1 h [36]. POD acts as a scavenger of toxic lipids and hydroperoxides generated from lipid peroxidation under Al stress [43]. Peroxidase also contributes to H_2_O_2_ detoxification by converting it into oxygen and water molecules [30]. In this study, the protein profile results showed that date palm callus with Al 200 mg.l^-1^ treatment responded by synthesising two new peptides with molecular weights of 19 and 29 KDa. A 42 KDa peptide disappeared, and a 29 KDa peptide appeared after the treatment with 150 mg.l^-1^ Al. These new peptides may have relevant roles in Al binding and may be low-molecular-weight proteins produced in plants as a response to abiotic stress [12]. The change in plant protein expression as detected by SDS PAGE after Al and other heavy metal stress was observed [2, 12, 44, 46].

## Conclusion

Our results highlighted for the first time the phytotoxic and genotoxic effect of Al at different concentrations (50, 100, 150 and 200 mg.l^-1^) on the *in vitro* cultures of date palm Hillawii cultivar. Generally, the growth and biochemical characteristics were reduced significantly after Al exposure. Al at 150 and 200 mg.l^-1^ decreased the fresh and dry weight and number of embryos in date palm callus compared with those in untreated ones. Total carbohydrates, total soluble proteins and free amino acids were also reduced significantly in the cultured tissues after Al treatments. MDA, H_2_O_2_ and peroxidase were increased in response to ROS generation in exposed calli. Genetic analysis by SDS-PAGE technique revealed that Al at 150 and 200 mg.l^-1^ induced the expression of the polypeptides of 29 and 19 KDa compared with that in the untreated ones. Our findings shed light on the importance of *in vitro* technique for further understanding of Al toxicity in date palm. Future research may be conducted to examine the effects of other Al concentrations on date palm cultured tissues and to evaluate genotoxicity. Furthermore, a tissue culture approach can be used to produce plants tolerant to Al toxicity.

## Data Availability

Not applicable.

## Abbreviations

FAA: free amino acid
GD: genetic distance
GS: genetic similarity
H_2_O_2_: Hydrogen Peroxide
KDa: kilo dalton
MDA: malondialdhyde
POD: peroxidase
ROS: reactive oxygen species
RPM: revolution per minute
RT: room temperature
SDS-PAGE: sodium dodecyl sulfate- polyacrylamide gel electrophoresis
UPGMA: unweighted pair group mean average
